# *Lysimachia* × *glabrophora* (Ericales, Primulaceae), a new wild nothospecies from the Republic of Korea

**DOI:** 10.3897/phytokeys.274.190054

**Published:** 2026-05-12

**Authors:** Kyu Tae Park, Jin Seok Kim, SeongJun Park, Minha Kim, Jeong-Ki Hong, KyoungSu Choi

**Affiliations:** 1 Department of Biology, College of Natural Sciences, Kyungpook National University, Daegu, 41566, Republic of Korea Bio-resource Research Center, Kyungpook National University Daegu Republic of Korea; 2 Bio-resource Research Center, Kyungpook National University, Daegu 41566, Republic of Korea College of Natural Sciences, Kyungpook National University Daegu Republic of Korea; 3 The Korean Plant Diversity Institute, Gimpo 10111, Republic of Korea The Korean Plant Diversity Institute Gimpo Republic of Korea; 4 Department of Life Sciences, Yeungnam University, Gyeongsan 38541, Republic of Korea Yeungnam University Gyeongsan Republic of Korea; 5 Division of Species Diversity Research Division, National Institute of Biological Resources, Incheon 22689, Republic of Korea National Institute of Biological Resources Incheon Republic of Korea; 6 Diversity Forecast & Evaluation Division, Nakdonggang National Institute of Biological Resources, Sangju 37242, Republic of Korea Nakdonggang National Institute of Biological Resources Sangju Republic of Korea

**Keywords:** East Asia, Lysimachia × glabrophora, molecular phylogenetics, morphology, natural hybridization, Republic of Korea

## Abstract

The genus *Lysimachia* (Primulaceae) is taxonomically complex, particularly within sect. *Spicatae* in East Asia. Field surveys in the Republic of Korea revealed a taxon exhibiting intermediate morphological states between *L. barystachys* and *L. fortunei*, including variation in leaf length-to-width ratio, raceme architecture, and floral density at the apex. Principal component analysis (PCA) of morphological traits positioned the putative hybrid between the parental taxa, forming a distinguishable cluster despite partial overlap. Phylogenetic analyses based on nrITS and plastome data revealed cytonuclear discordance, consistent with a hybrid origin. Integrating morphological and molecular evidence, this taxon is described as a new wild nothospecies, Lysimachia × glabrophora.

## Introduction

The genus *Lysimachia* comprises approximately 280 species (POWO 2026), distributed mainly across temperate and subtropical regions of the Northern Hemisphere, with a center of diversity in East Asia (Hu and Kelso 1996; Takhtajan 1997; Mabberley 2008). Most members of the genus are perennial or annual herbs, although some are subshrubs or aquatics (Hu and Kelso 1996). This wide morphological variation and ecological breadth have made *Lysimachia* one of the most taxonomically complex genera in Primulaceae, particularly in species delimitation and sectional classification (Anderberg et al. 2007).

Currently, the genus *Lysimachia* is divided into six subgenera: Heterostylandra (Hand.-Mazz.) F.H.Chen & C.M.Hu, Idiophyton Hand.-Mazz., *Lysimachia* L., *Lysimachiopsis* A.Heller, *Naumburgia* (Moench) Klatt, and Palladia (Moench) Hand.-Mazz. (Chen and Hu 1979; Hao et al. 2004). Within subgenus Palladia, sect. *Spicatae* is of particular interest. Although geographically restricted and relatively species-poor, this section is taxonomically significant for understanding diversification in East Asia (Chen and Hu 1979; Kim et al. 2007). Sect. *Spicatae* includes several species such as *L. clethroides* Duby, *L. barystachys* Bunge, and *L. fortunei* Maxim., which are distinguished by their characteristic spike-like inflorescences (Kim et al. 2007). In Korea, the presence of sect. *Spicatae* has been documented, and earlier morphological studies have examined relationships among *L. clethroides*, *L. barystachys*, and *L. fortunei* (Kim et al. 2007). Subsequent molecular studies have confirmed their status as distinct taxa (Hao et al. 2004; Wang et al. 2016; Liu et al. 2023; Yan and Hao 2024).

Natural hybrids have been reported in Lysimachia, including L. × pilophora, L. × radfordii, L. × intermedia, L. × producta, and L. × commixta. Among these, L. × pilophora, occurring in Japan, has been documented as a natural hybrid between *L. clethroides* and *L. fortunei* within sect. *Spicatae* (POWO 2026). Such reports suggest that hybridization contributes to morphological intermediacy and taxonomic complexity in the genus.

Chloroplast DNA has long been employed in phylogenetic studies of angiosperms because it is uniparentally inherited, structurally conserved, and useful for resolving relationships at intergeneric and interspecific levels (Chen et al. 2023; Guan et al. 2024). In contrast, nuclear DNA regions such as the internal transcribed spacer (ITS) region and low-copy nuclear genes evolve more rapidly, often revealing variation at or below the species level. Nuclear DNA is also biparentally inherited, enabling the detection of reticulate evolutionary processes such as hybridization and polyploidy (Lee et al. 2022). Therefore, the combined use of plastome and ITS data is particularly effective for identifying hybrid origins in *Lysimachia*.

During field surveys in Korea, populations showing morphological intermediacy between *L. barystachys* and *L. fortunei* were identified. These individuals could not be clearly assigned to any known species, suggesting a potential hybrid origin. In this study, the hybrid status of this distinct taxon is evaluated using plastome and nrITS data, and a formal description of a new wild nothospecies, Lysimachia × glabrophora, is provided.

Materials and methods

Specimens of the putative hybrid were collected from Toerae-ri, Hallim-myeon, Gimhae-si, Gyeongsangnam-do, Republic of Korea, between 2023 and 2025. Qualitative traits examined included leaf shape, arrangement, margin type, apex, and base form; presence of hairs on adaxial and abaxial leaf surfaces; stem pubescence and branching pattern; indumentum of inflorescences, bracts, and pedicels; and calyx shape. The examined quantitative traits included leaf length, leaf width, inflorescence length, bract length, pedicel length, calyx lobe length, number of flowers per 20 mm of inflorescence at the base and apex, flower density at the base and apex of the inflorescence, and flowering time. All measurements were taken with digital calipers. Traits were compared with specimens of morphologically similar congeners (*L. clethroides*, *L. barystachys*, and *L. fortunei*) from the KNU, KWNU, and KHB herbaria to evaluate diagnostic differences. These traits were selected because they are recognized as key diagnostic characters for distinguishing species within sect. *Spicatae* (Kim et al. 2007).

Principal component analysis (PCA) was performed on 70 individuals to assess the morphological intermediacy of the putative hybrid. The analysis was based on multiple quantitative traits (Table 2), encompassing both vegetative and reproductive characters. Differences among taxa were further evaluated using one-way analysis of variance (ANOVA), followed by Tukey’s HSD test for multiple comparisons when significant differences were detected. All statistical analyses were conducted in R.

Total genomic DNA was extracted using two methods tailored to specific downstream applications. A CTAB-based protocol (Doyle and Doyle 1987) was used for samples intended for NGS analysis to maximize yield, while the DNeasy Plant Mini Kit (Qiagen, Valencia, CA, USA) was employed for ITS sequencing following the manufacturer’s instructions. For plastome sequencing, high-quality DNA was used to construct an SMRTbell library, which was sequenced on the PacBio Revio platform (Pacific Biosciences, Menlo Park, CA, USA). To obtain nuclear sequence data, the nrITS region was amplified using primers ITS4 and ITS5 (White et al. 1990), and the PCR products were sequenced by Sanger sequencing.

Assembly of the complete L. × glabrophora plastome was performed using Hifiasm v0.19.9-r616 with default parameters optimized for high-fidelity long-read data (Cheng et al. 2021). Contigs were assembled in Geneious Prime (https://www.geneious.com) using similarity searches against the reference plastome of *L. clethroides* (LC758799). Circularity was confirmed by identifying overlapping sequences at contig ends, and structural integrity was validated by remapping HiFi reads onto the consensus sequence. Protein-coding genes (PCGs) were annotated with GeSeq (Tillich et al. 2017), while transfer RNA (tRNA) genes were validated using tRNAscan-SE v2.0 (Chan et al. 2021). Annotation accuracy was checked against reference plastomes of *Lysimachia* species available in GenBank (Table 1).

**Table 1.** Accessions of *Lysimachia* and related taxa examined in this study.

Species	Plastome accession number	ITS accession number
*Primula poissonii*	NC_024543	HM018204
*Embelia vestita*	MN167884	HG004815
*Ardisia crenata*	MW929178	FJ482136
*L. monelli*	LC758814	MG877752
*L. laxa*	LC758780	JF976948
*L. microcarpa*	LC758776	MG877813
*L. foenum-graecum*	LC758788	MG877787
*L. capillipes*	LC758781	MG877765
*L. vulgaris*	LC758795	AF547736
*L. davurica*	LC758805	MG877777
*L. longipes*	LC758806	JF976952
*L. fortunei*	LC758799	MG877789
*L. clethroides*	LC758782	JF976897
*L. barystachys*	LC758794	MG877763
**L. × glabrophora**	PX512378	PX563291, PX563292
*L. decurrens*	LC758785	MG877778
*L. lobelioides*	LC758793	JF976950
*L. heterogenea*	LC758773	JF976938
*L. mauritiana*	NC_060700	KF954521
*L. pentapetala*	LC758801	MG877823
*L. stenosepala*	LC758809	MH808420
*L. glanduliflora*	LC758778	MG877791
*L. auriculata*	LC758791	MG877762
*L. circaeoides*	LC758808	MG877772
*L. maritima*	MW029425	MG877758
*L. alfredii*	LC758783	JN638405
*L. patungensis*	LC758797	JF976964
*L. klattiana*	LC758774	MG877803
*L. fordiana*	LC758784	MG877788
*L. paridiformis* var. *stenophylla*	LC758802	MG877820
*L. grammica*	LC758800	MG877793
*L. pseudohenryi*	-	MG877828
*L. japonica*	LC758796	MG877802
*L. chekiangensis*	LC758807	MG877767
*L. hemsleyana*	NC_052863	JF976928
*L. fistulosa* var. *wulingensis*	LC758786	MG877786
*L. hemsleyi*	LC758779	MG877795
*L. rubiginosa*	LC758811	JF976972
*L. congestiflora*	LC758798	JF976900
*L. melampyroides*	LC758810	JF976956
*L. deltoidea* var. *cinerascens*	LC758792	JF976909
*L. phyllocephala*	LC758803	JF976968
*L. hypericoides*	LC758790	-
*L. fukienensis*	LC758787	-
*L. parvifolia*	LC758775	-

The systematic placement of L. × glabrophora was determined using two datasets: nrITS and 75 plastome PCGs (Table 1). In addition, nrITS sequences were identified from the HiFi dataset through BLAST searches against ITS reference sequences. Matched reads were extracted and examined individually to identify distinct ITS variants. Multiple ITS haplotypes were reconstructed based on sequence variation and subsequently aligned using MAFFT v.7.222 (Katoh and Standley 2013) with default parameters. To investigate potential reticulate evolutionary patterns, a phylogenetic network was constructed in SplitsTree v.6.4.7 (Huson and Bryant 2006) using the aligned dataset.

Seventy-five PCGs were selected based on their consistent presence across all taxa. *Primula poissonii* was used as the outgroup, following previous phylogenomic studies of *Lysimachia* plastomes (Liu et al. 2023), as it is a closely related member within Primulaceae that provides robust rooting for the phylogenetic tree. For each dataset, multiple sequence alignments were performed using MAFFT v7.222 (Katoh and Standley 2013), and poorly aligned regions were removed using Gblocks v0.91b (Talavera and Castresana 2007), retaining approximately 99% of the original sequences.

Phylogenetic trees were reconstructed using both maximum likelihood (ML) and Bayesian inference (BI) approaches. Optimal substitution models were identified with ModelFinder in IQ-TREE v2.1.2 (Nguyen et al. 2014) under the Bayesian Information Criterion (BIC). The GTR+F+I+R3 model was selected for the plastome dataset, while the GTR+I+G4 model was applied to the ITS dataset. ML analyses were conducted in IQ-TREE v2.1.2 with 1,000 bootstrap replicates to assess node support. BI analyses were performed in MrBayes v3.2.7 (Ronquist et al. 2012) with 1,000,000 generations, sampling every 1,000 generations, and discarding the first 25% of trees as burn-in.

## Results

### Morphological observations

Lysimachia × glabrophora is morphologically intermediate between *L. barystachys* and *L. fortunei*, exhibiting a combination of vegetative and reproductive traits from both taxa. The leaf length-to-width ratio of L. × glabrophora ranges from 2.3 to 5.4, overlapping with *L. fortunei* (2.1–4.4) and *L. barystachys* (2.9–8.1) (Table 2). It shares glabrous leaves and stems with *L. fortunei*, whereas *L. barystachys* is characterized by multicellular hairs. In contrast, L. × glabrophora differs from *L. fortunei* in having narrower leaves and a more compact inflorescence structure. Floral density at the apex in L. × glabrophora ranges from 10 to 24 flowers per 20 mm, denser than the lax arrangement in *L. fortunei* (5–10) but overlapping with *L. barystachys* (21–32). The number of flowers per raceme (8–12) and internode length (1.3–4.9 mm) also show intermediate or overlapping ranges relative to *L. barystachys* and *L. fortunei*.

**Table 2.** Qualitative and quantitative morphological characters of Lysimachia × glabrophora and related species in sect. *Spicatae*.

Character	*L. barystachys*	*L. clethroides*	*L. fortunei*	L. × glabrophora
Habitat	Mountain, grassy mountain slopes	Damp woodland margins, roadsides	Wetland	Disturbed lowland habitats
Leaf				
Arrangement	Alternate	Alternate	Alternate	Alternate
Shape	Oblong-lanceolate to oblanceolate	Narrowly elliptic to broadly lanceolate	Oblong to lanceolate	Oblong to lanceolate
Margin	Acute	Acuminate	Short acuminate	Acute
Base	Attenuate	Cuneate	Cuneate	Cuneate
Length (mm)	47.0–(64.0)–79.3	69.4–(122.96)–154.3	47.3–(59.5)–72.7	33.5–(64.6)–79.2
Width (mm)	9.5–(12.07)–15.3	20.4–(38.10)–57.9	11.4–(15.8)–23.3	8.1–(12.0)–14.8
Length-to-width ratio	2.9–(5.5)–8.1	1.7–(3.29)–4.3	2.1–(2.8)–4.4	2.3–(5.4)–9.8
Adaxial hairs	Multicellular, long	Glabrous/nearly glabrous	Glabrous	Glabrous
Abaxial hairs	Multicellular, long	Glabrous/nearly glabrous	Glabrous	Glabrous
Stem				
Hair	Multicellular-long hair	Multicellular	Glabrescent	Glabrous
Branching	Unbranched	Unbranched	Branched	Branched
Diameter (mm)	3.0–(4.5)–6.0	2.24–(2.98)–4.21	1.5–(2.4)–3.2	2.5–(3.5)–4.5
Inflorescence				
Shape	Gooseneck or erect (rare)	Gooseneck or erect (rare)	Erect	Erect or gooseneck
Type	Densely flowered raceme	Densely flowered raceme	Lax raceme	Moderately flowered raceme
Hair	Glandular or multicellular	Glandular	Glabrous	Glabrous
Length (mm)	47.3–(93.8)–142.8	58.7–(129.4)–219.0	59.7–(129.4)–200.4	67.2–(140.6)–257.5
Flowers				
Flowers per 20 mm (base)	8–(12.6)–15	6–(8.7)–13	5–(8.0)–10	8–(9.9)–12
Internodes per 20 mm (base)	2.0–(3.2)–3.9	1.3–(2.7)–4.5	2.1–(3.9)–5.7	1.3–(2.7)–4.9
Flowers per 20 mm (apex)	21–(26.4)–32	13–(18.8)–26	10–(17.5)–28	10–(17.6)–24
Internodes per 20 mm (apex)	1.0–(1.1)–1.2	0–(1.0)–1.8	0.8–(1.6)–2.6	0.5–(1.2)–1.9
Pedicel length (mm)	2.3–(4.3)–7.4	2.0–(4.6)–7.4	1.3–(2.9)–4.1	2.1–(3.1)–4.8
Pedicel hairs	Pubescent	Pubescent	Glabrous	Glabrous
Petal length (mm)	3.9–(5.3)–6.0	3.3–(6.5)–8.7	2.5–(2.9)–4.2	2.8–(2.9)–4.0
Petal color	White	White	White	White
Calyx length (mm)	1.2–(3.0)–5.5	1.3–(2.2)–3.1	1.2–(3.0)–7.1	1.4–(2.2)–3.4
Calyx hairs	Pubescent	Pubescent	Glabrous	Glabrous
Bract length (mm)	5.1–(7.7)–12.2	2.5–(6.7)–11.3	1.1–(3.4)–4.9	3.1–(6.8)–9.3
Flowering time	June–August	June–August	July–August	July–August

**Figure 1.**
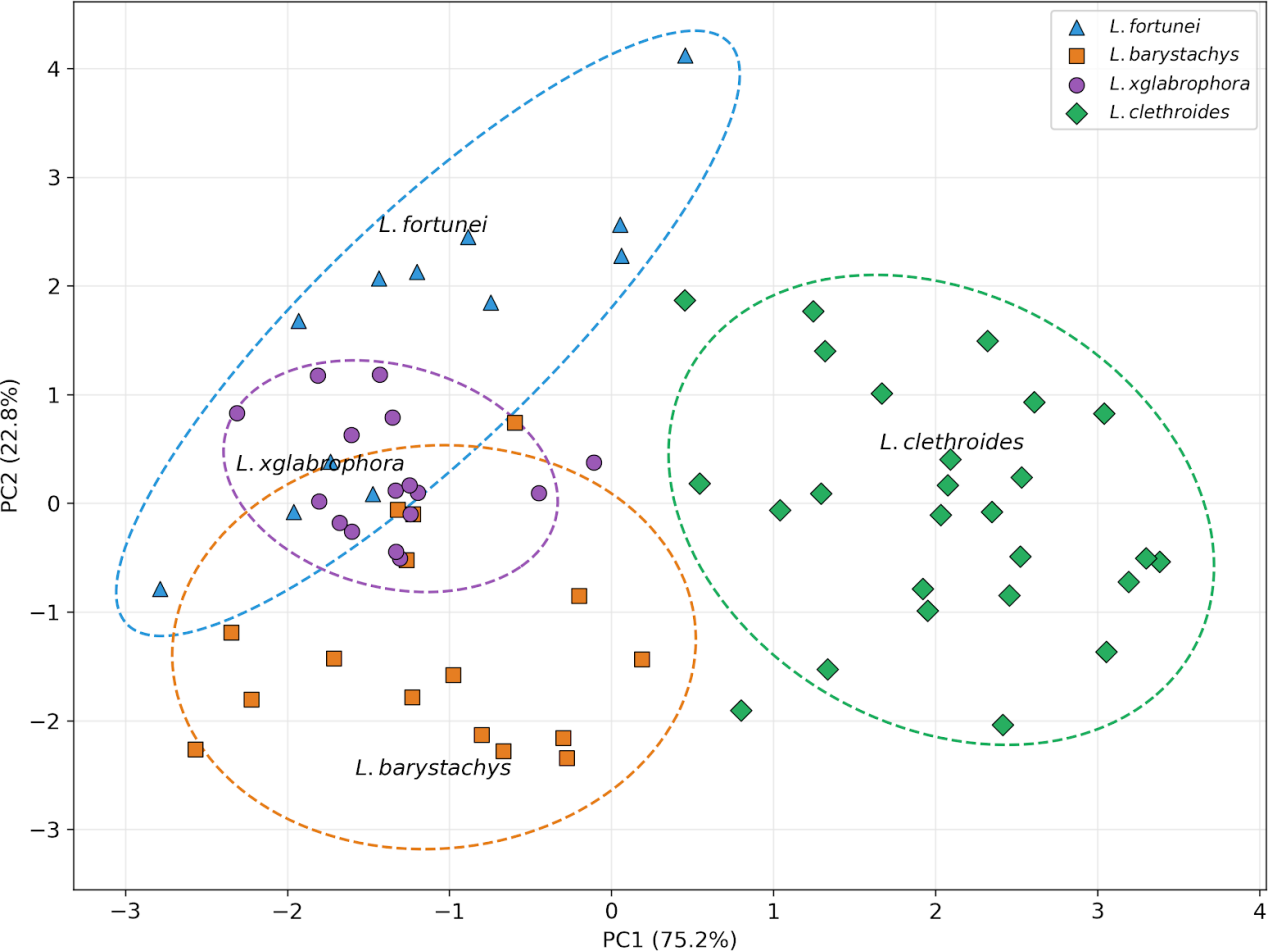
Principal component analysis (PCA) of quantitative morphological traits in Lysimachia × glabrophora, *L. barystachys*, and *L. fortunei*. Axes represent PC1 and PC2, which together explain the majority of variation.

PCA based on multiple quantitative traits demonstrated that L. × glabrophora occupies an intermediate position between *L. barystachys* and *L. fortunei*, with partial overlap with both taxa (Fig. 1). The first two principal components explained a large proportion of the total variation, and the three taxa were distributed along a continuous gradient rather than forming completely distinct clusters. Separation along PC1 and PC2 reflected the combined variation in multiple morphological traits, particularly those related to leaf morphology and inflorescence structure.

**Figure 2.**
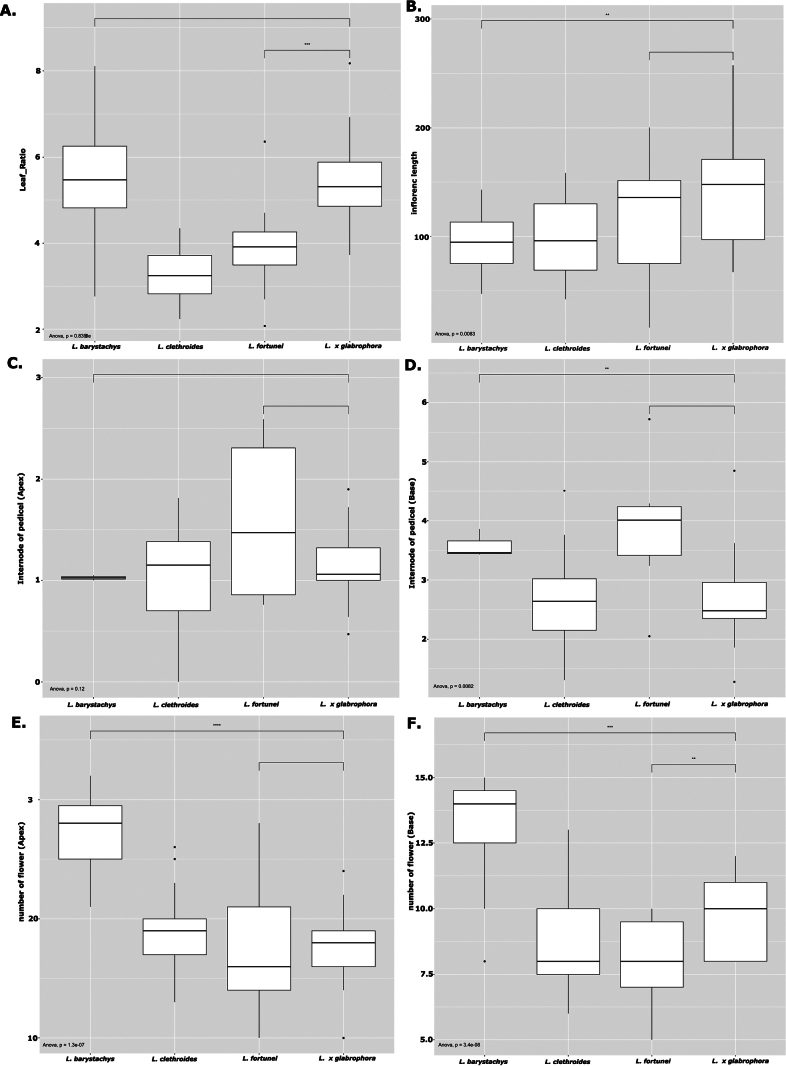
Boxplots showing variation in quantitative morphological traits among *L. barystachys*, *L. clethroides*, *L. fortunei*, and L. × glabrophora. Differences among taxa were assessed using one-way ANOVA. Asterisks indicate levels of statistical significance (* *P* < 0.05, ** *P* < 0.01, *** *P* < 0.001).

ANOVA revealed significant differences among taxa for several quantitative traits (Fig. 2), although not all measured characters showed statistically significant variation. Post hoc comparisons further supported differentiation among taxa in multiple traits, while L. × glabrophora retained overlapping ranges with both *L. barystachys* and *L. fortunei*.

### Plastome features

**Figure 3.**
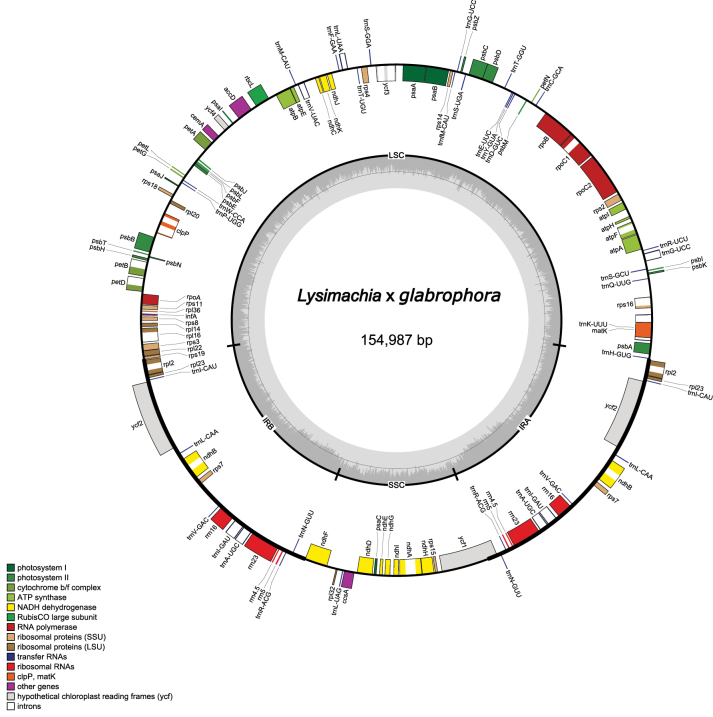
Plastome map of Lysimachia × glabrophora. Genes transcribed clockwise are shown outside the circle, while those transcribed counterclockwise are shown inside. Genes are color-coded by functional group.

The plastome of L. × glabrophora (Fig. 3) is 154,987 bp in length. It exhibits the typical quadripartite organization, consisting of two inverted repeats (IRs) (25,976 bp each) separating the large single-copy (LSC; 85,021 bp) and small single-copy (SSC; 18,104 bp) regions. The overall GC content is 37.0%. The genome encodes 112 unique genes, including 79 PCGs, 29 tRNA genes, and four rRNA genes.

### Phylogenetic position

**Figure 4.**
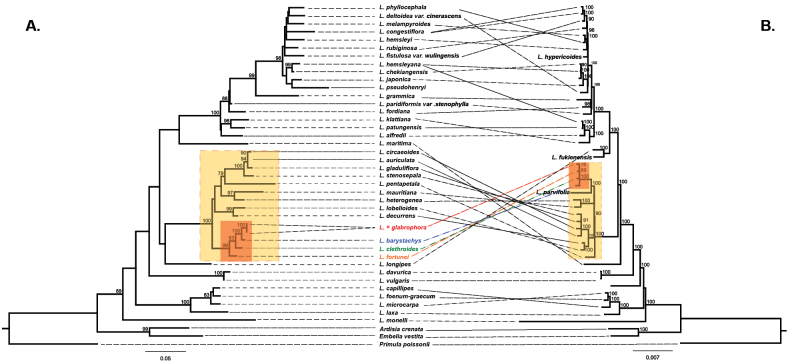
Phylogenetic relationships of *Lysimachia* species inferred from nrITS sequences (**A**) and 75 plastome protein-coding sequences (**B**). Numbers above branches indicate bootstrap support (BS) values. Yellow boxes denote subgenus *Palladia*; red boxes highlight sect. *Spicatae*.

Two ML trees were reconstructed using the nrITS region and 75 plastome PCG sequences to clarify the phylogenetic placement of L. × glabrophora (Fig. 4). Both datasets produced broadly consistent topologies, with most major clades of *Lysimachia* strongly supported (bootstrap ≥ 90).

However, several incongruences were evident between the nuclear and plastid trees. In the nrITS tree, L. × glabrophora was placed as a sister taxon to *L. barystachys*, while *L. clethroides* and *L. fortunei* formed separate lineages (Fig. 4A). In contrast, in the plastome tree, L. × glabrophora was nested within a clade comprising *L. barystachys*, *L. clethroides*, and *L. fortunei*, clustering most closely with *L. fortunei* (Fig. 4B). Overall, both phylogenies consistently identified close relationships among L. × glabrophora and sect. *Spicatae* species (*L. barystachys*, *L. clethroides*, and *L. fortunei*) and supported the monophyly of the sect., although the exact placement of L. × glabrophora differed between the two datasets.

**Figure 5.**
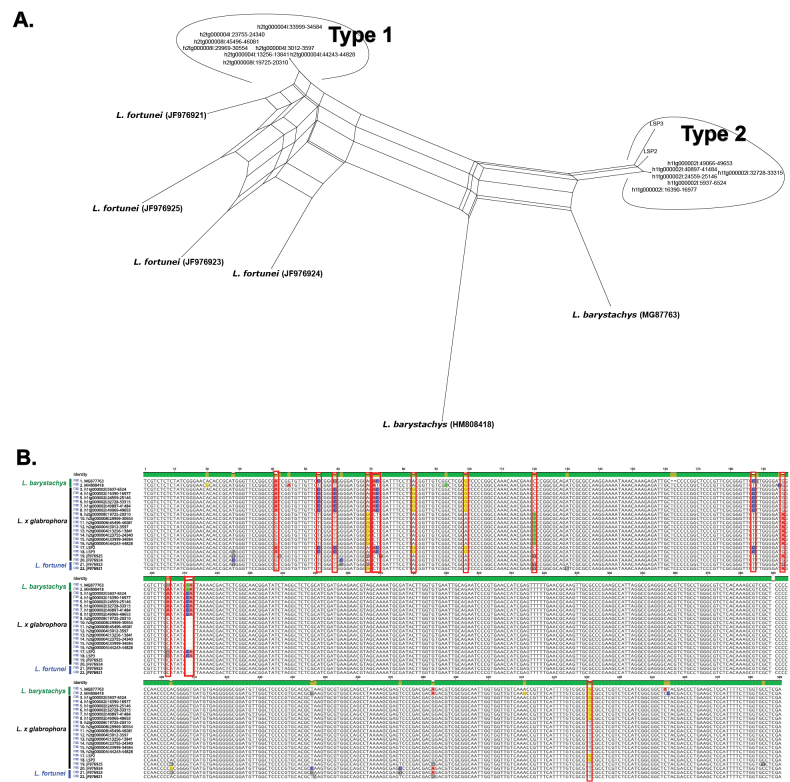
**A**. Phylogenetic network based on nrITS sequences constructed in SplitsTree, showing relationships among *L. barystachys*, *L. fortunei*, and L. × glabrophora. Two distinct ITS haplotypes (Type 1 and Type 2) were identified in L. × glabrophora, showing affinities to *L. fortunei* and *L. barystachys*, respectively; **B**. Alignment of nrITS sequences highlighting polymorphic sites among taxa, indicating intra-individual variation in L. × glabrophora.

Analysis of nrITS sequences revealed the presence of multiple ITS variants within L. × glabrophora (Fig. 5). These variants were resolved into two distinct haplotypes (Type 1 and Type 2), showing affinities to *L. fortunei* and *L. barystachys*, respectively. Sequence alignment further revealed several polymorphic sites within L. × glabrophora, consistent with intra-individual variation in ITS sequences.

### Taxonomic treatment

#### Lysimachia × glabrophora

Taxon classificationPlantaeEricalesPrimulaceae

J.S.Kim & K.Choi, notho sp. nov.

1D9072DE-651A-5324-86CE-AC73CBA4E2B6

urn:lsid:ipni.org:names:77379930-1

Fig. 6

##### Type.

Republic of Korea, • Gyeongsangnam-do, Gimhae-Si, Hallim-myeon, 35°18'33"N, 128°47'52"E, 10 August 2023, *Choi KS & Kim JS 20230810* (holotype: KNU!, isotypes: KB!, NNH!).

**Figure 6.**
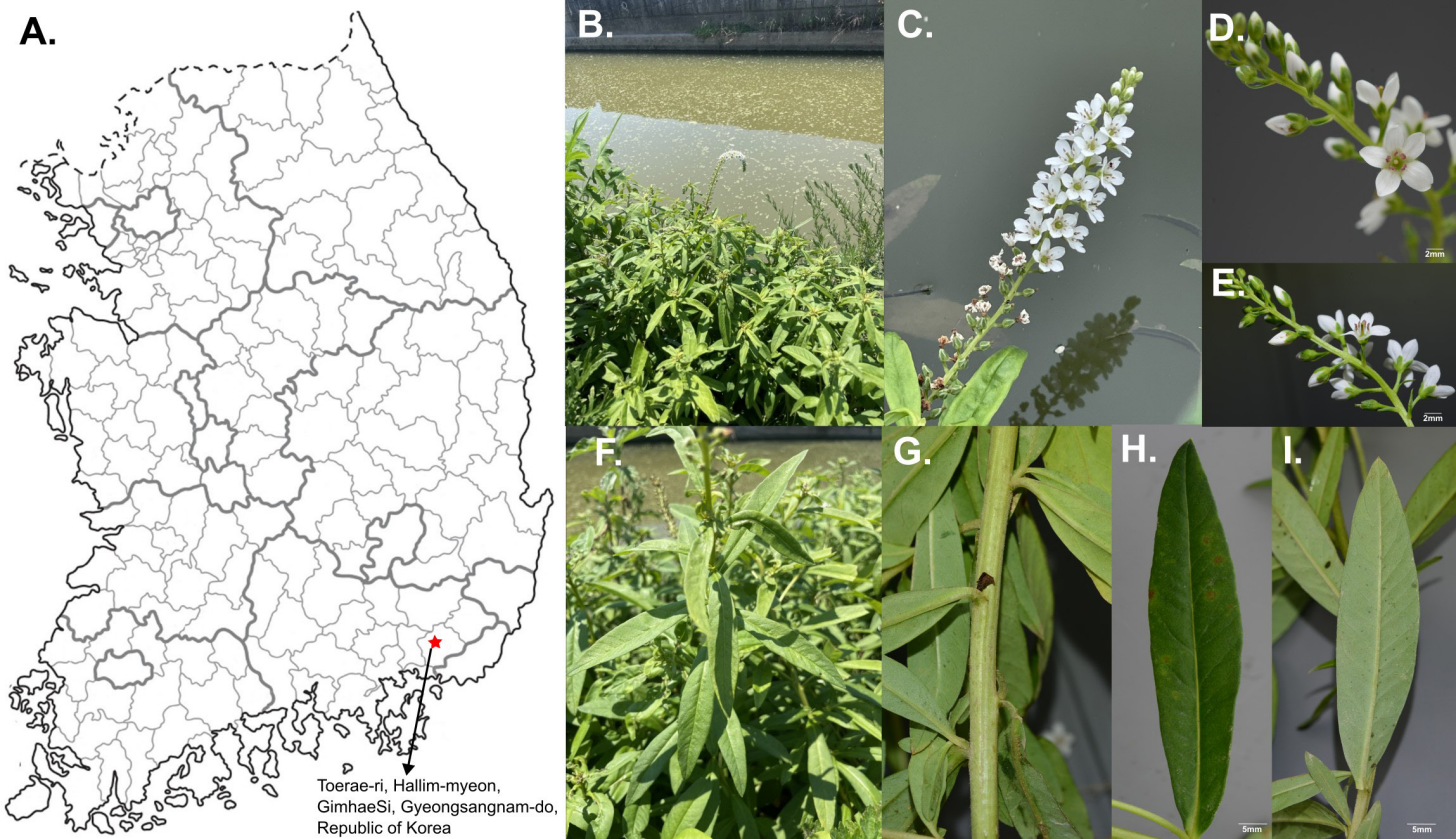
Morphological characteristics of Lysimachia × glabrophora. **A**. Location; **B**. Habitat; **C**. Inflorescence; **D**. Flowers; **E**. Stamens and stigma; **F**. Leaf; **G**. Stem; **H**. Adaxial leaf surface; **I**. Abaxial leaf surface. Photos by J.S. Kim and K. Choi.

##### Diagnosis.

**Figure 7.**
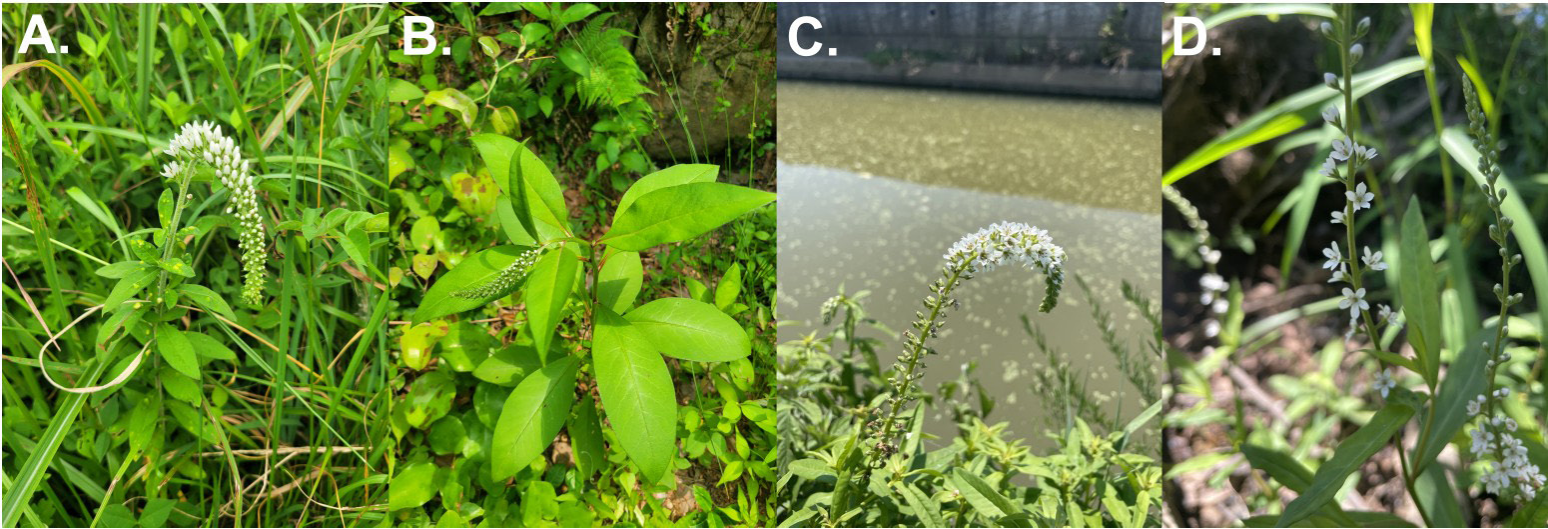
Comparison of inflorescences among four *Lysimachia* species. **A**. *L. clethroides*; **B**. *L. barystachys*; **C**. *L.* × *glabrophora*; **D**. *L. fortunei*. Photos by S. Park and K. Choi.

Lysimachia × glabrophora can be distinguished from closely related species in sect. *Spicatae* by a combination of vegetative and inflorescence traits. It differs from *L. barystachys* in having glabrous stems and erect racemes, whereas *L. barystachys* is characterized by hairy stems and typically curved (gooseneck) racemes. In comparison with *L. clethroides*, it is distinguished by unbranched peduncles and narrower, oblong to lanceolate leaves, while *L. clethroides* usually bears branched peduncles. It is further distinguished from *L. fortunei* by its relatively narrower leaves and racemes with intermediate floral density, compared to the broader leaves and more densely flowered racemes of *L. fortunei* (Fig. 7).

##### Description.

Perennial herb, 60–90 cm tall, with long-creeping rhizomes. Stems erect, 2.5–4.5 mm in diameter, circular in cross-section, glabrous, simple or branched in the upper part; lower portions often tinged reddish. Leaves alternate, sessile or subsessile, oblong to lanceolate, 33.5–79.2 × 8.1–14.8 mm; length-to-width ratio 3.7–8.2; base cuneate; margins entire; apex acute to acuminate; surfaces glabrous; venation pinnate, secondary veins inconspicuous. Inflorescences terminal racemes, erect to gooseneck-shaped, 67.3–257.5 mm long; rachis glabrous. Floral density: 8–12 flowers per 20 mm at the base, 10–24 flowers at the apex. Pedicels 2.2–4.9 mm long, glabrous; internodes 1.3–4.9 mm at the base, 0.5–1.9 mm at the apex. Bracts lanceolate, 3.2–9.4 mm long, glabrous. Calyx lobes 5, 1.4–3.4 mm long, glabrous. Petal lobes 5, white, 2.7–4.0 mm long; stamens 5, adnate to the corolla base; filaments free. Ovary superior, 1-locular; style filiform; stigma capitate.

##### Etymology.

The epithet *glabrophora* is derived from the Latin *glaber* (“hairless”) and the Greek *-phora* (“bearing”), referring to the predominantly glabrous stems, inflorescences, and filaments.

##### Phenology.

Flowering observed from July to August.

##### Distribution and habit.

Lysimachia × glabrophora occurs in Hallim-myeon, Gimhae-Si, Gyeongsangnam-do, Republic of Korea (Fig. 6A). It grows naturally in disturbed lowland habitats, particularly between cultivated fields and irrigation channels.

##### Vernacular name.

Keun-jin-peo-ri-kka-chi-su-yeom (큰진퍼리까치수염).

## Discussion

Morphological and molecular evidence together provide insight into the taxonomic position of the putative hybrid L. × glabrophora. Morphologically, the species exhibits traits that are intermediate between *L. barystachys* and *L. fortunei*. Its leaves are narrowly oblong to lanceolate, broader than those of *L. barystachys* but narrower and more acute than those of *L. fortunei*. Both adaxial and abaxial surfaces are glabrous, resembling *L. fortunei*, whereas *L. barystachys* typically bears multicellular hairs. Inflorescences are intermediate, with floral density greater than that of *L. fortunei* but less than that of *L. barystachys* or *L. clethroides* (Fig. 7).

Kim et al. (2007) reported that *L. clethroides* and *L. barystachys* are more closely related to each other than to *L. fortunei*, based on qualitative traits. The morphological observations in this study are consistent with that interpretation. Examination of KH herbarium specimens from the same locality revealed collections previously identified as *L. fortunei*. Detailed re-examination showed that L. × glabrophora shares several traits with *L. fortunei* (glabrous leaves, stems, and inflorescences) but differs in floral arrangement. Specifically, L. × glabrophora exhibits a significantly higher floral density at the apex (10–24 flowers per 20 mm), whereas *L. fortunei* has a laxer arrangement (Table 2). Differences in leaf shape (length-to-width ratio 3.73–8.17) and branching of the inflorescence axis further distinguish the two taxa. PCA results (Fig. 1) support its morphological distinctiveness.

Quantitative analyses reinforce this interpretation. One-way ANOVA revealed significant differences among taxa for several quantitative morphological traits, including leaf morphology and inflorescence-related characters (Fig. 3). Post hoc comparisons indicated that L. × glabrophora differs from *L. fortunei* in floral density and from *L. barystachys* in leaf morphology, while retaining overlapping ranges with both taxa. These results suggest that L. × glabrophora exhibits a distinct yet intermediate morphological pattern.

Multivariate analysis based on PCA provides additional support for its putative hybrid origin (Fig. 1). In morphospace, L. × glabrophora occupies an intermediate position between *L. barystachys* and *L. fortunei*, reflecting its intermediate inflorescence architecture and flower density. Molecular evidence further corroborates this interpretation. Phylogenetic analysis of the nrITS region places L. × glabrophora in close affinity with *L. barystachys*, whereas plastid gene analysis clusters it with *L. fortunei*. This cytonuclear discordance is consistent with a hybrid origin (Fig. 4). In addition, HiFi sequencing revealed multiple ITS variants within L. × glabrophora (Fig. 5), which were resolved into distinct haplotypes showing affinities to *L. barystachys* and *L. fortunei*. This intra-individual variation further supports the interpretation of a putative hybrid origin.

Natural hybridization has been documented in *Lysimachia*, including L. × pilophora, a hybrid between *L. clethroides* and *L. fortunei* within sect. *Spicatae*, indicating that such processes are not uncommon in the genus. The occurrence of L. × glabrophora, together with herbarium records, suggests that *L. barystachys* and *L. fortunei* may overlap in distribution, providing opportunities for natural hybridization. These findings imply that hybridization may play an important role in generating morphological diversity within sect. *Spicatae*.

Taken together, the morphological intermediacy, multivariate analyses, and molecular evidence obtained in this study support a hybrid origin of L. × glabrophora. Although additional data would be valuable to confirm its exact parentage, current evidence justifies recognition of L. × glabrophora as a putative hybrid between *L. barystachys* and *L. fortunei*.

## Supplementary Material

XML Treatment for Lysimachia × glabrophora

## Additional information

### Artificial Intelligence (AI) use

The authors accept full responsibility for the content of the manuscript, including the disclosure of any use of AI.

No AI tools were used in the preparation of this manuscript.

### Author ORCIDs

K. T. Park https://orcid.org/0000-0002-9640-6683

J. S. Kim https://orcid.org/0000-0002-4474-1244

S. Park https://orcid.org/0009-0007-0058-8077

M. Kim https://orcid.org/0009-0006-9203-7944

J.-K. Hong https://orcid.org/0000-0002-5450-8331

K. Choi https://orcid.org/0000-0003-4879-3161

### Data availability

The two newly sequenced ITS sequences (accession numbers: PX563291 and PX563292) and plastome sequence (accession number: PX512378) have been submitted to the NCBI. The voucher specimens of Lysimachia × glabrophora were deposited at the herbarium of Kyungpook National University (Daegu, Republic of Korea).
